# Supplementary material for: High-Throughput Site-Specific N-Glycosylation Profiling of Human Fibrinogen in Atrial Fibrillation
Source: J Proteome Res. 2025 Mar 18;24(4):2121–34. doi: 10.1021/acs.jproteome.5c00096 (PMC11976851; doi:10.1021/acs.jproteome.5c00096)
Supplement: Supplementary file 1 — pr5c00096_si_001.pdf [file pr5c00096_si_001.pdf]

Supporting information – Figures

High-Throughput Site-Specific N-Glycosylation Profiling of Human Fibrinogen in Atrial Fibrillation

Dinko Šoić[1], Domagoj Kifer[1], Janko Szavits-Nossan[2,3,4], Aleksandar Blivajs[5], Lovorka Đerek[6], Diana Rudan[5], Olga Gornik[1], Ivan Gudelj[7*], Toma Keser[1*]

[1] Faculty of Pharmacy and Biochemistry, University of Zagreb, Ante Kovačića 1, 10000 Zagreb, Croatia

[2] Magdalena University Hospital for Cardiovascular Diseases, Radnička cesta 32, 10000 Zagreb, Croatia

[3] Faculty of Dental Medicine and Health, J.J. Strossmayer University in Osijek, Crkvena 21, 31000 Osijek, Croatia

[4] Faculty of Medicine, J.J. Strossmayer University of Osijek, Josipa Huttlera 4, 31000 Osijek, Croatia

[5] Department of Cardiology, University Hospital Dubrava, Avenija Gojka Šuška 6, 10000 Zagreb, Croatia

[6] Clinical Department for Laboratory Diagnostics, University Hospital Dubrava, Avenija Gojka Šuška 6, 10000 Zagreb, Croatia

[7] Faculty of Biotechnology and Drug Development, University of Rijeka, Radmile Matejčić 2, 51000 Rijeka, Croatia

* Correspondence:

Toma Keser toma.keser@pharma.unizg.hr

Ivan Gudelj ivan.gudelj@biotech.uniri.hr

Supplementary Figures S1-S3

Figure S1. Absolute intensity of the most abundant fibrinogen glycopeptide peak, N4H5S1 $[M+3H]^{3+}$, across different peptides, measured from 20 µL of human plasma precipitated with 10%, 15%, and 20% absolute ethanol.

Figure S2. MS/MS fragmentation spectra of N4H5S2 glycoforms from both fibrinogen glycosylation sites**.** *A*, fragmentation pattern of Beta.normal-N4H5S2 $[M+3H]^{3+}$ glycopeptide. *B*, fragmentation pattern of Gamma.MISS-N4H5S2 $[M+3H]^{3+}$ glycopeptide.

Figure S3. MS/MS fragmentation spectra of minor glycoforms from the fibrinogen Gamma glycosylation site**.** *A*, fragmentation pattern of Gamma.MISS-N4H5 $[M+3H]^{3+}$ glycopeptide. *B*, fragmentation pattern of Gamma.MISS-N4H5S1F1 $[M+3H]^{3+}$ glycopeptide. *C,* fragmentation pattern of Gamma.MISS-N4H5S2F1 $[M+3H]^{3+}$ glycopeptide.

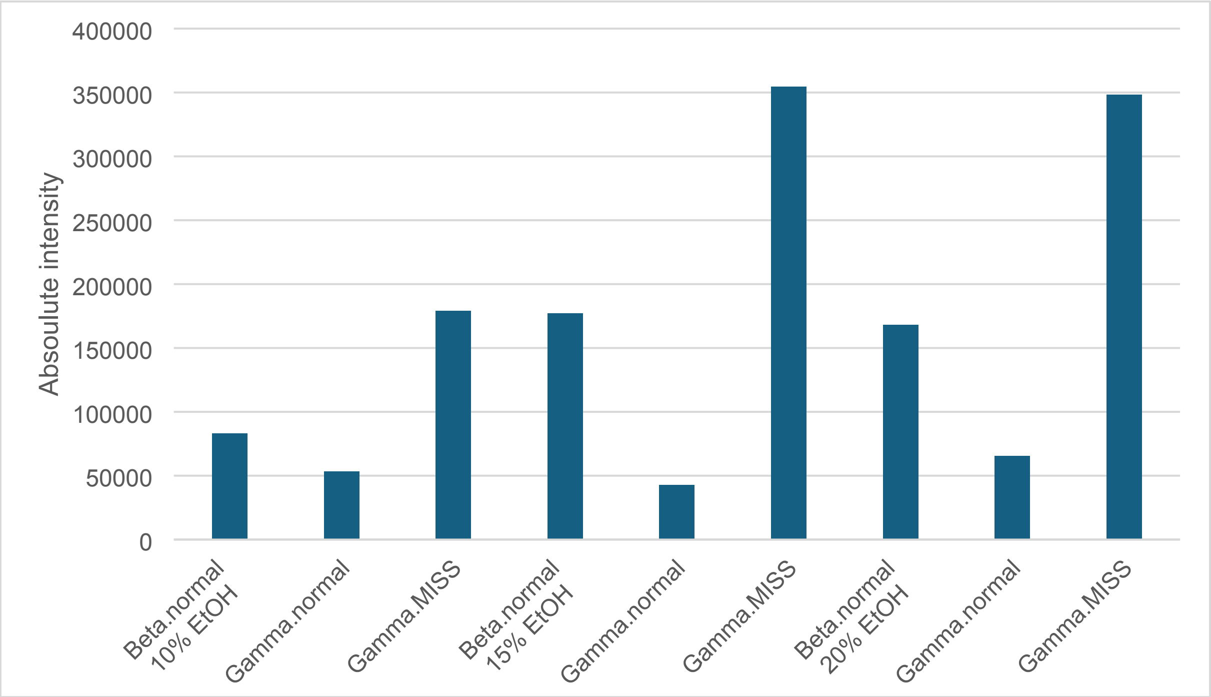

Figure S1. Absolute intensity of the most abundant fibrinogen glycopeptide peak, N4H5S1 $[M+3H]^{3+}$, across different peptides, measured from 20 µL of human plasma precipitated with 10%, 15%, and 20% absolute ethanol.

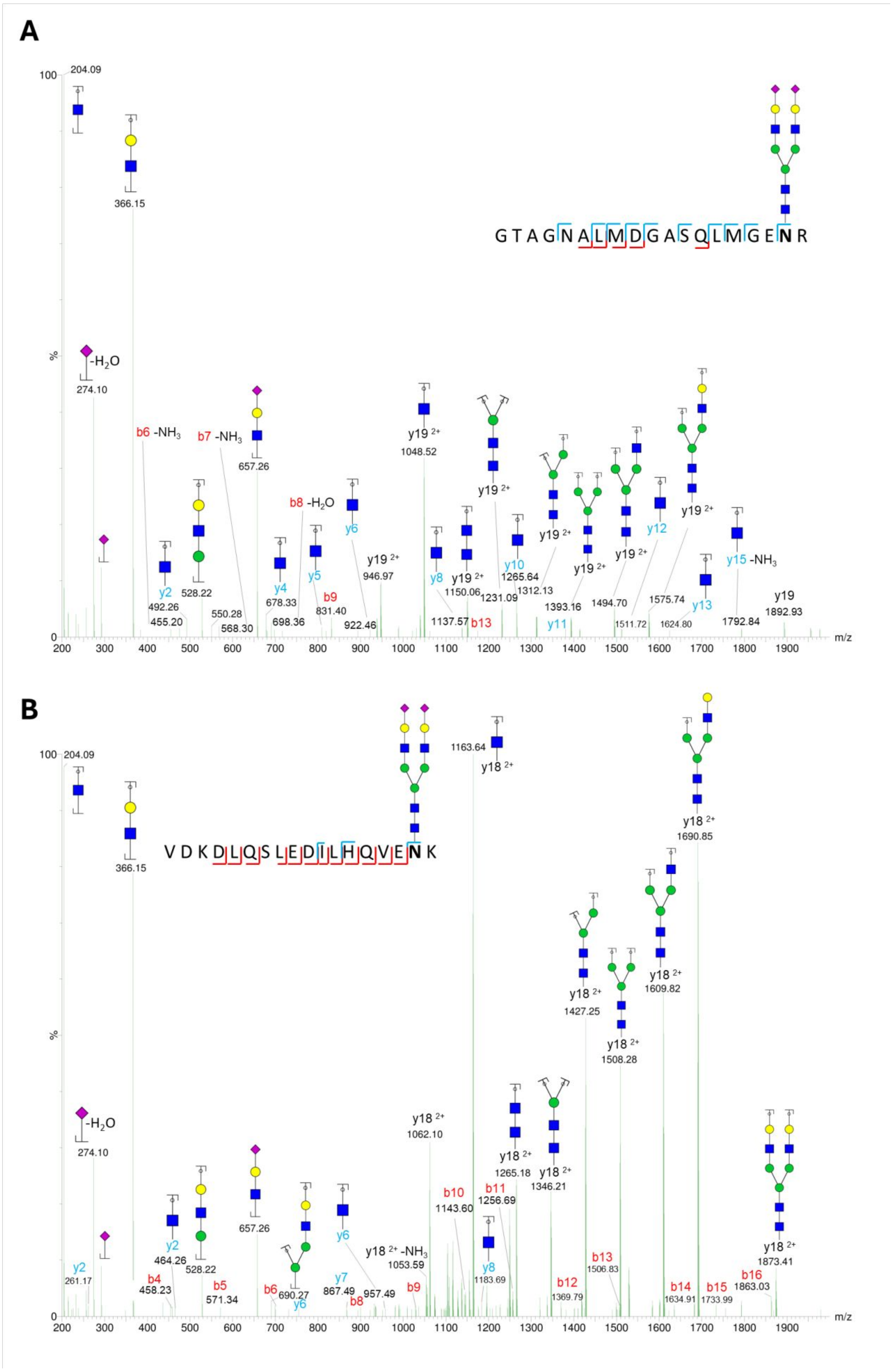

Figure S2. MS/MS fragmentation spectra of N4H5S2 glycoforms from both fibrinogen glycosylation sites**.** *A*, fragmentation pattern of Beta.normal-N4H5S2 $[M+3H]^{3+}$ glycopeptide. *B*, fragmentation pattern of Gamma.MISS-N4H5S2 $[M+3H]^{3+}$ glycopeptide.

A

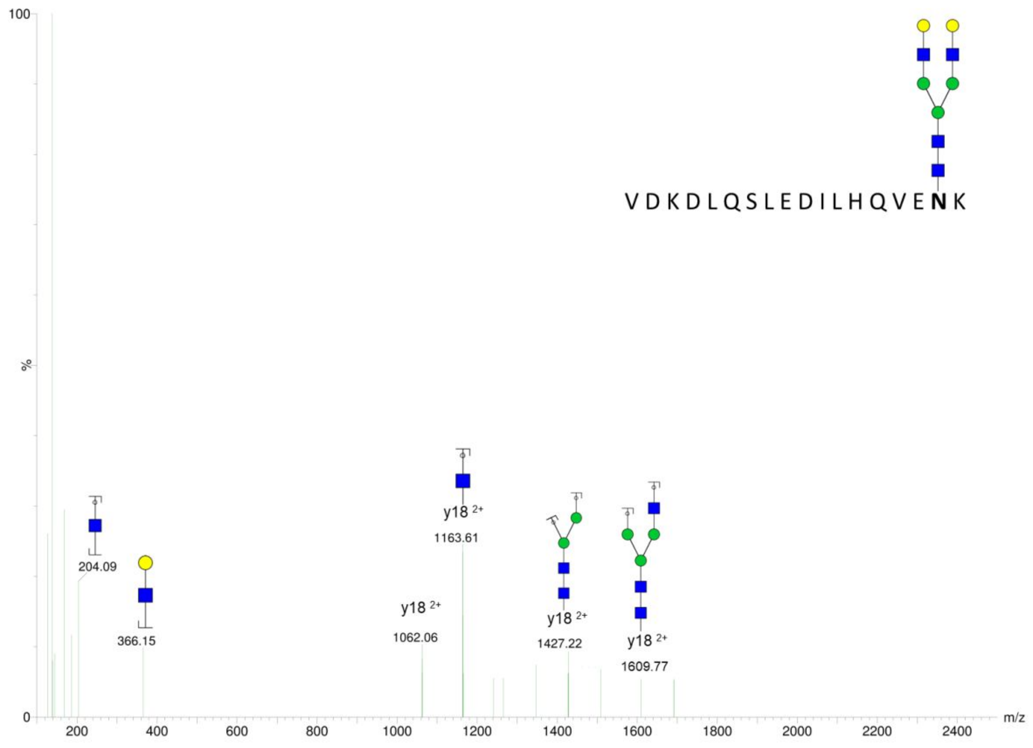

VDKDLQSLEDILHQVENK
100
%
0
204.09
366.15
y18 2+
1163.61
y18 2+
1062.06
y18 2+
1427.22
y18 2+
1609.77
m/z
200
400
600
800
1000
1200
1400
1600
1800
2000
2200
2400

B

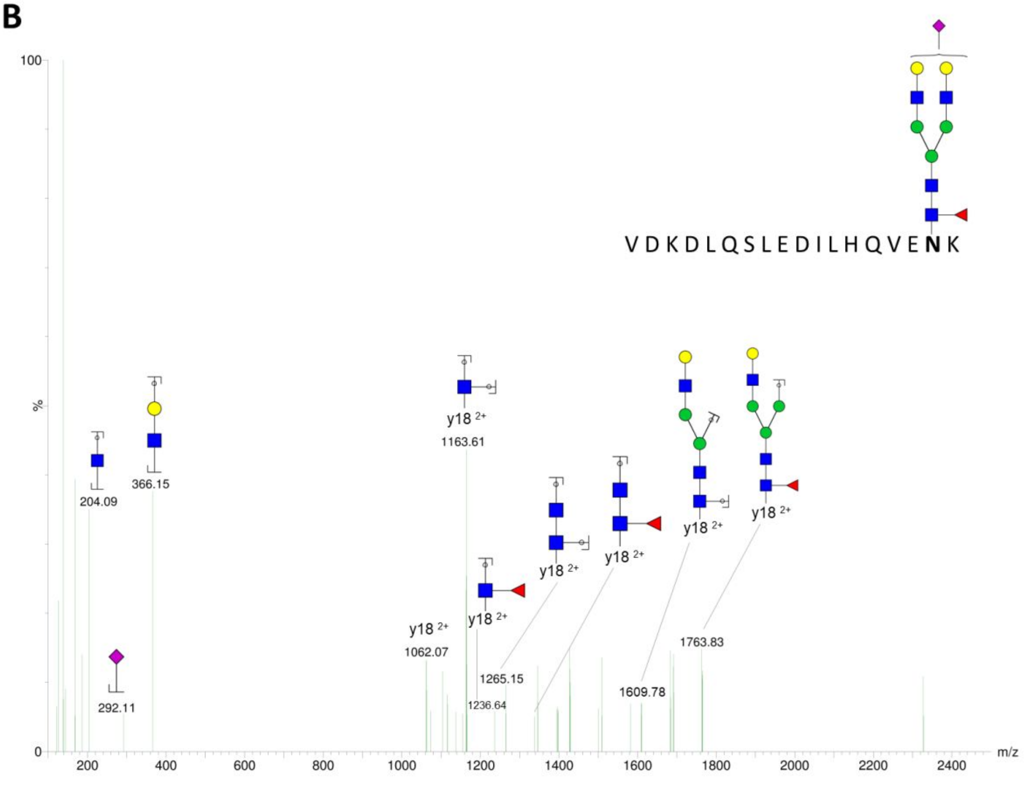

VDKDLQSLEDILHQVENK
100
%
0
204.09
366.15
292.11
y18 2+
1163.61
y18 2+
1062.07
y18 2+
1236.64
y18 2+
1265.15
y18 2+
1609.78
y18 2+
1763.83
m/z
200
400
600
800
1000
1200
1400
1600
1800
2000
2200
2400

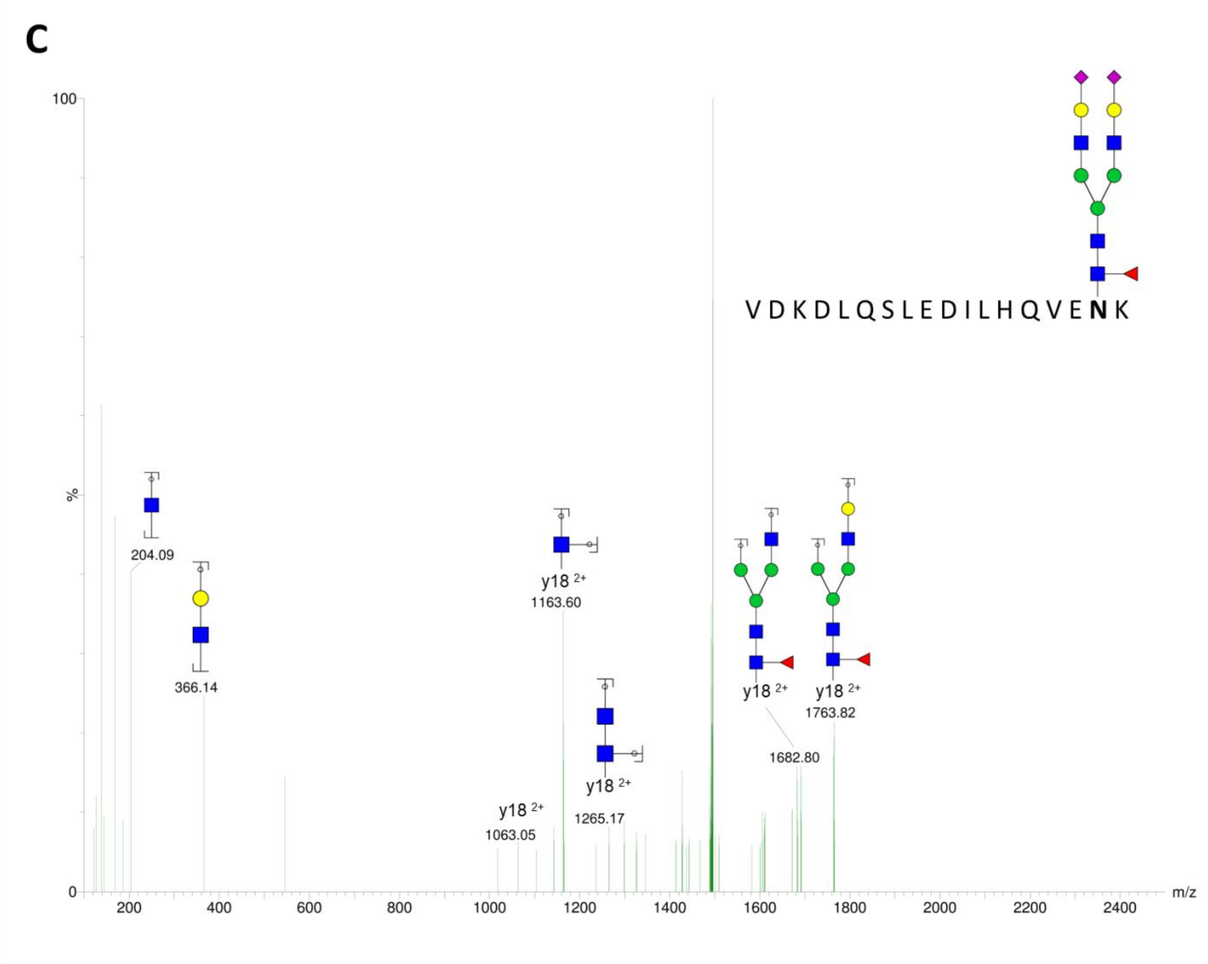

Figure S3. MS/MS fragmentation spectra of minor glycoforms from the fibrinogen Gamma glycosylation site**.** *A*, fragmentation pattern of Gamma.MISS-N4H5 $[M+3H]^{3+}$ glycopeptide. *B*, fragmentation pattern of Gamma.MISS-N4H5S1F1 $[M+3H]^{3+}$ glycopeptide. *C,* fragmentation pattern of Gamma.MISS-N4H5S2F1 $[M+3H]^{3+}$ glycopeptide.
